# Community health workers: to train or to restrain? A longitudinal survey to assess the impact of training community health workers in the Bolama Region, Guinea-Bissau

**DOI:** 10.1186/1478-4491-12-8

**Published:** 2014-02-11

**Authors:** Sérgio C Lopes, António J Cabral, Bruno de Sousa

**Affiliations:** 1 The MENTOR Initiative, Angola; 2 CMDT, Center for Malaria and Tropical Medicine, Lisbon, Portugal; 3 Faculdade de Psicologia e Ciências da Educação, Universidade de Coimbra, Coimbra, Portugal

**Keywords:** Community health workers, Training, Impact, Diagnosis accuracy, Diarrheal diseases

## Abstract

**Background:**

The shortage in human resources for health affects most dramatically developing countries which frequently use community health workers (CHW) as the basis for health programmes and services. The traditional definition refers CHWs as members of the community who are recruited and trained in health prevention and promotion to provide services within their community. In Guinea-Bissau, CHWs play a fundamental role in the diagnosis and treatment of childhood diarrheal diseases - one of the main health problems in the country.

**Methods:**

This study is based on 22 CHW, 79% of the total number of CHW in the Sanitary Region of Bolama. The main goal was to assess how training CHW on diarrheal diseases impacted the accuracy of the diagnosis and treatment of these diseases in children under the age of 5 years. Three evaluations were made throughout time - one evaluation before the training and two follow-up evaluations.

An observation grid was developed to evaluate the identified signs, symptoms, diagnosis and treatments prescribed by the CHW in consultations to children with a suspicion of diarrhoeal disease. A similar grid was filled by a medical doctor who took the role of the external validation standard.

Friedman’s variance analysis and Cochran’s Q test were performed to compare the accuracy depicted by CHWs in identifying items throughout time. A logistic regression model was also used to check the possible influence of socio-demographic characteristics of CHWs on the accuracy of the diagnosis and treatment prescribed by the CHW.

**Results:**

The results show that CHWs improve significantly their performance in identifying the correct diagnosis in the first follow-up moment after the training (*P* = 0.001, *n* = 22) but, 3 months later, the effectiveness decreases. No statistical evidence was found for the logistic regression models applied.

This progressive loss of performance after training may occur because CHWs fail to apply treatment algorithms and guidelines over time.

A limited set of socio-demographic characteristics of the CHWs can influence their performance and should not be disregarded when selecting CHW candidates.

**Conclusion:**

The selection, supervision, support and continuous training of CHW are as important as the training provided.

## Background

Health systems are typically unbalanced as the quantity and quality of services tend to be inversely correlated with the needs felt by the population [1], affecting mainly the poorer and rural populations and, more generally, those who face accessibility barriers to health care [2-4]. Since infrastructure, health technologies and human resources tend to be concentrated in urban centres, people living in poorer and rural areas tend to have less access to health care, despite their greater needs [5].

This unequal resource distribution is frequent in low-income countries, where the chronic shortage of human resources for health (HRH) limits the provision of health care in the most remote rural areas. The African continent, for example, only avails 3% of the world HRH, despite enduring 24% of the global burden of disease [3].

As human resources are even less available in remote rural areas, many health programmes in low-income countries started to use community health workers (CHW) as providers of health services. Typically, these CHWs are members of the community who are recruited and trained in health prevention and promotion to provide services within their community [6]. CHWs represent an important group among health workers in low-income countries; in Africa, they comprise almost one-third of the total health workforce [5].

The evaluation of their performance shows mixed results. Certain health programmes which have used CHWs claimed significant improvements in infant mortality and morbidity rates for some major infant diseases [7], while others reported no significant impact on the health status of their communities, with their quality of the health care being rated as mediocre [8].

### CHW training and supervision

Training is a core factor to CHWs’ performance, as well as supervision, technical and material support [9]. CHW’s training varies in time, methods and continuity after the initial instruction. The majority of programmes offer annual refresher courses to review concepts and practice [10]. Some authors defend that 3 days of additional annual training improve the quality of services provided [11], whereas abilities and knowledge are quickly lost if the refresher courses are not made available [9].

Along with the experience in CHW programmes, training is now becoming more practical and focusing on competence acquisition [9], although the evaluation of training is still commonly focused on theory and not on acquired practical competences. In Senegal and Bolivia, training provided improved CHW theoretical competences in respiratory diseases management, but practical competences were not assessed [12,13]. Ashwell and Freeman noted that CHWs in a Papua-New Guinea province had good levels of theoretical knowledge, but only 15 out of 22 had clinical competence. They also demonstrated that the competence of rural CHWs was worse, due to lack of supervision and support [14].

Supervision and support are considered crucial to the success of programmes using CHWs [15,16]. However, many programmes do not consider supervision and support a priority because of its cost, and this may contribute to poor results [9]. Supervision is also important in the very remote areas because it counters isolation and helps to motivate and engage CHWs in their tasks [9,11].

### Guinea-Bissau’s context, health system and health problems

Guinea-Bissau is one of the poorest countries in the world, rated as 164th among the 169 countries in the Human Development Index (2010), with 65.7% of the population living on less than 1 USD/day [17].

The health system in Guinea-Bissau shows great asymmetries, because many of the facilities damaged during the civil war of 1998 to 1999 have not been rehabilitated [18], and because HRH are scarce and unevenly distributed: 51% of health professionals are working in the Bissau region, 59% of physicians are allocated to referral hospitals, while only 24% work in primary health care [19].

The basic health units (BHU) are the local facilities in rural areas, where care is provided by local CHWs and midwives. In 2011, at the time of the study, these workers provided minimal curative and preventive care, but they were not recognized as active members of the national health system. The health policy at the time recommended that the numbers of CHWs should not increase, but their quality and performance should be improved [19].

In Guinea-Bissau, it is estimated that 223 out of 1,000 children die before the age of 5 years. Infant mortality rates in urban areas are lower than in rural areas, with 106/1,000 deaths against 150/1,000, respectively [19]. In 2010, Malaria, diarrheal diseases and respiratory infections represented 55% of all causes of death for children aged under 5 years, with diarrheal diseases alone being responsible for 19% of these deaths [20].

The Health Region of Bolama (HRB) is one of the 11 Health Regions in the country, where health care in the remote villages was provided by 28 CHWs (in 2011) providing preventive and curative care for most common diseases. Diarrhoea is one of the diseases that CHWs were trained to diagnose and treat at the rural BHUs in the Region.

By improving health coverage, CHWs are seen as a very cost-effective resource, because they provide health care (at a lower cost) to a large number of people who previously had no access to health care at all [8,21]. However, on equity, quality and effectiveness grounds, it becomes important to evaluate how this care is provided and what factors affect service outcomes [3]. CHW programmes’ implementation must balance economical advantages with a continuous concern of programmes’ effectiveness and quality of care provided.

Reaching out to CHWs can be an effective alternative when facing the scarcity of health workers in Guinea-Bissau, as long as there is evidence of their added value in the proper treatment of some of the main health problems in remote rural areas. This study aims to evaluate the short-term impact of training in the CHWs’ performance on diagnosing and treating diarrheal diseases in children aged under 5 years, and the evolution on diagnosis accuracy at 1 and 3 months after training.

## Methods

### Study design

A quantitative approach was used to measure the impact of training on the performance of CHWs. A longitudinal survey was conducted consisting of three different periods - before training on diarrhoeal diseases management has been provided, and 1 and 3 months after the training occurred. The main objectives were to check variations in CHWs’ capacity to diagnose and treat diarrheal diseases and to assess the impact of training on CHWs’ performance at the two selected moments after training.

Data were registered every time that a diagnosis of diarrhoea was suspected in children younger than 5 years of age brought to the CHWs for care. All cases were collected in BHU where CHWs usually provide care. At each moment of the evaluation, five different medical consultations were recorded for each CHW.

### Study population

All CHWs from the HRB were considered as potential participants. Two of them refused to participate and four were considered lost to follow-up: a total of 22 CHWs out of 28 participated in the study, five women and 17 men. They were between 19 and 56 years old and the majority were subsistence farmers (14). One CHW had 11 years of completed school education, while two had never gone to school. Half of the CHWs had completed between 4 to 6 years of schooling.

Fifteen CHWs had less than 10 years of experience and the remaining had between 15 to 25 years of experience as a CHW. All the CHWs had had at least one training on diarrhoeal diseases management. Five of them, the oldest ones, had had 10 trainings on the subject.

### Data collection

We considered the training provided to CHWs as the independent variable, while the measured dependent variable was defined as diagnosis and treatment accuracy (DTA). Given that there is no formal definition of DTA, Last’s [22] definitions were used to define conceptually the dependent variable. Therefore, DTA was considered as the capacity to successfully determine an individual health status and consequently prescribe the correct treatment.

WHO/UNICEF [23] guidelines on diarrhoeal diseases management were used to create an evaluation grid applied to the collection of the data. Guidelines consider three steps to provide a good treatment: Signs and Symptoms, Diagnosis, and Treatments. These steps were considered as the component variables of the main variable. Taking into account the DTA’s definition above, it was assumed that CHWs followed these guidelines, they could effectively diagnose and treat diarrhoeal diseases.

In the first step - Signs and Symptoms - CHWs should have done the clinical history and should objectively assess the presence/absence of the following items: Diarrhoea starting date; Number of daily dejections; Blood in the stool; Lethargy or unconsciousness; Restlessness or irritability; Sunken eyes; Drinking poorly or not able to drink; Drinking eagerly or thirsty; Very slow skin retraction after pinching (>2 s). For the second step - Diagnosis - five items were included in the grid: Severe dehydration; Acute Dehydration; No dehydration; Chronicle dehydration; Dysentery. In the last step - Treatments - the grid had three hypotheses: Oral hydration with water; ORS hydration; send to Hospital.

The grid used in this survey was adapted from the original guidelines, after consideration of the local reality and the opinion of medical experts. These experts were part of the NGO medical team who was providing training to CHW. Taking in account their field experience, they analyzed the WHO/UNICEF grid and selected the items which were part of training programme and part of CHW practical competences. As the objective was to assess CHWs’ accuracy, the grid should only assess what CHW were able to do in their day-to-day practice. A total of five items from the initial grid based on WHO/UNICEF’s guidelines were removed from the final grid applied in the study - Persistent Chronic Diarrhoea in Diagnosis and EV Fuids, Antibiothics, Multivitamins and Zinc in Treatments - as the CHWs were not allowed to prescribe these treatments.

For each child aged under 5 years with suspicion of diarrhoea, the CHW made a consultation identifying Signs and Symptoms, Diagnosis and Treatments items. An observer collected and registered the procedures undertaken by the CHW in the observation grids. These registered observations were later compared with the report made by a medical doctor, who was considered as the verification standard. Items were considered as correctly identified if they were a match to the doctor’s identification. To increase the grid’s feasibility, data were always collected by the same person using the same criteria. It was also based on rigid guidelines, using simple items hardly subject to dubious interpretations.

A questionnaire was applied to identify some CHWs socio-demographic characteristics, such as age, sex, civil status, number of completed school years, profession, place of work, number of years as a CHW, number of diarrhoeal diseases training courses completed. The scores of theoretical knowledge on diarrhoeal diseases were obtained from the training evaluation forms. Pre- and post-training questionnaires were applied by the trainers and the scores were used in the study to characterize the CHWs’ levels of knowledge.

### Data analysis

The initial analysis explored trends in the utilization of the guidelines for Signs and Symptoms, Treatments and Diagnosis. Four different levels of measuring accuracy were considered. For Signs and Symptoms and Treatments, the analysis considered three levels of matching of results between CHWs and the ‘verification standard’ (the medical doctor):

1. Total of items correctly identified in the three moments - Total of matching items identified by the CHW and the medical doctor;

2. Correctly identified items by the CHW in the three moments when both CHW and medical doctor identified these items as present and/or needed in a child;

3. Correctly identified items by the CHW in the three moments when both CHW and medical doctor identified these items as neither present nor needed in the child.

For the Diagnosis, only one possibility was considered, that is

4. The items that were correctly identified by both the CHW and the medical doctor.

Data analysis was performed in order to check for statistically significant differences between the three moments in the study. Due to data limitations, two non-parametric tests were considered: Friedman’s variance test and Cochrane’s Q test.

Friedman’s variance test was carried out in order to check for variations throughout time in the median scores of success in identification of items. Multiple comparisons were performed every time the null hypothesis of equality of the median scores was rejected.

In the Cochrane’s Q test, data were grouped by medical consultation in order to better assess differences. In other words, all first consultations from all CHWs in the first moment were compared with all first consultations from all CHWs in the second and third moment (represented in Results as C1), and so on until all five consultations were compared in the three moments (represented in Results as C2, C3, C4 and C5). Proportions of correct answers were compared and multiple comparisons were also made to check which transitions had statistically significant differences.

In addition, a logistic regression (LR) model was used to test the relationship between CHWs’ socio-demographic characteristics and the scores and evolution of their diagnosis and treatment accuracy. The application of the LR model has been restricted to those moments where statistically significant differences were found by the Cochran’s Q tests.

The full set of statistical analysis was made using SPSS 19.0 software, and considering a significance level of 5%.

### Ethical issues

The study was approved by the Bolama’s Regional Health Board. All participants were previously informed of the study’s objectives and methods, and those who agreed to participate gave their written informed consent.

## Results and discussion

### Results

A common trend was found across the various forms of analysis of data: we have observed an improvement in correct item identification from moment 1 (before the training) to moment 2 (1 month after training), followed by a decline from moment 2 to moment 3 (3 months after training).

Results are grouped by Signs and Symptoms, Diagnosis and Treatments. For each item, the exploratory data analysis, Friedman’s variance and Cochran’s Q test results are presented. The last part is a summary of the results obtained from the LR model applied.

### Signs and symptoms

A trend of improvement in correct identification from the first moment to the second (before and after training) was observed, followed by a decline from the second to the third moment (1 and 3 months after training). Three items did not follow this tendency (Drinking poorly or not able to drink; Drinking eagerly or thirsty). Six out of nine items consistently displayed a correct identification rate below 80% across the three moments of the evaluation, for some of their identification signs.

Significant statistical differences were verified in four items: Number of days (*P* = 0.005, *n* = 22); Number of daily dejections (*P* = 0.002, *n* = 22); Sunken eyes (*P* = 0.018, *n* = 22); and Drinking eagerly or thirsty (*P* = 0.012, *n* = 22). Multiple comparisons showed significant improvements from moment 1 to moment 2 for the item Number of daily dejections (*P* = 0.016, *n* = 22) and a significant decrease in the correct identification of signs from moment 2 to moment 3 for the item Number of days (*P* = 0.048, *n* = 22).

**Figure 1:**
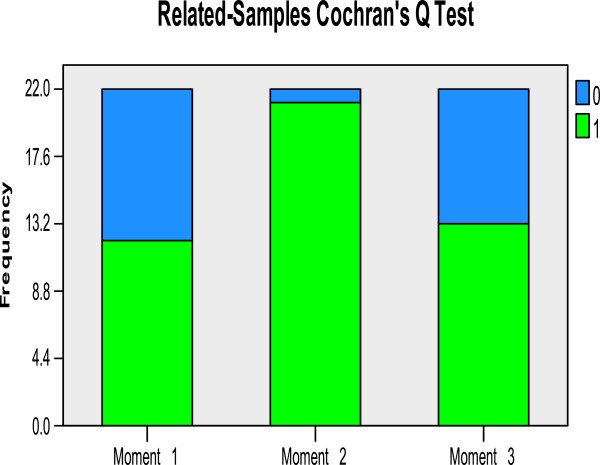
**Cochran’s Q test results to item number of diary dejections: success proportions over the three moments, Bolama Region, Guinea**-**Bissau, 2011.** Legend: Success Proportion Variation over the three moments of evaluation obtained by performing Cochran’s Q test, where 1 means successful and 0 means unsuccessful.

The Cochran’s Q test results displayed similar trends. Multiple comparisons demonstrate a statistically significant rise in the proportion of correct identification from the first to the second moment for items Number of daily dejections (*P* = 0.003, *n* = 22 – Figure 1) and Sunken eyes (*P* = 0.013, *n* = 22). The transition from the second to the third moment registered a significant decrease in two items: Number of days (*P* = 0.008, *n* = 22); and Lethargy*/*unconsciousness (*P* = 0.008, *n* = 22).

### Diagnosis

**Figure 2:**
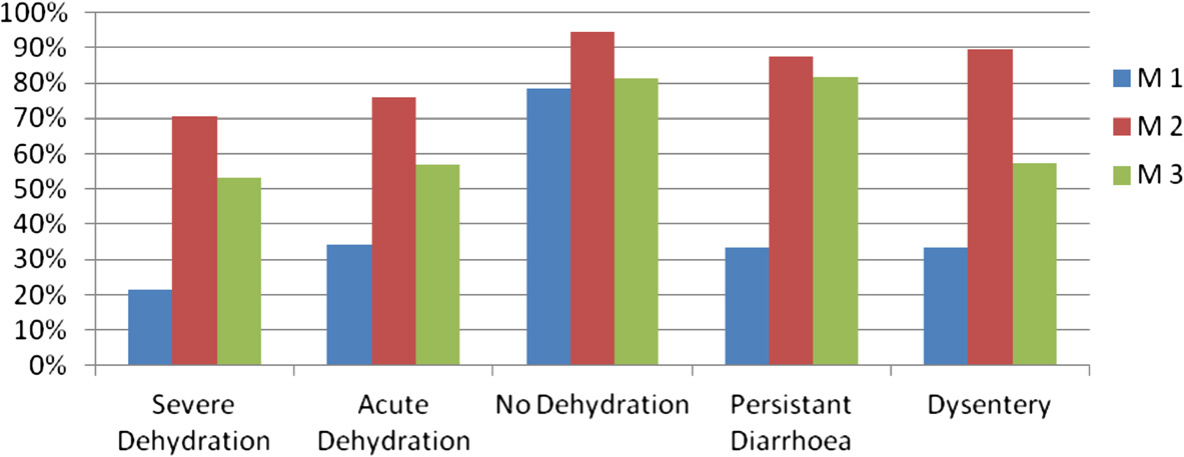
**Percentage of diagnosis correctly identified by CHWs over the three moments, Bolama Region, Guinea**-**Bissau, 2011.**

Apart from diagnostic No dehydration, all diagnosis had an identification success rate below 35% in the first moment. Figure 2 outlines the impact that training had on CHWs’ performance from the first to the second moment. Analysis also stated that CHWs confuse mostly two types of diagnosis: Severe dehydration and Acute dehydration. In the first moment, 57% of the total cases of Severe dehydration were misdiagnosed as Acute dehydration, and 31% of the cases of Acute dehydration were misdiagnosed as Severe dehydration cases. This problem improved partially immediately after training, but 3 months later similar ratios of incorrectly diagnosed cases were observed.

**Figure 3:**
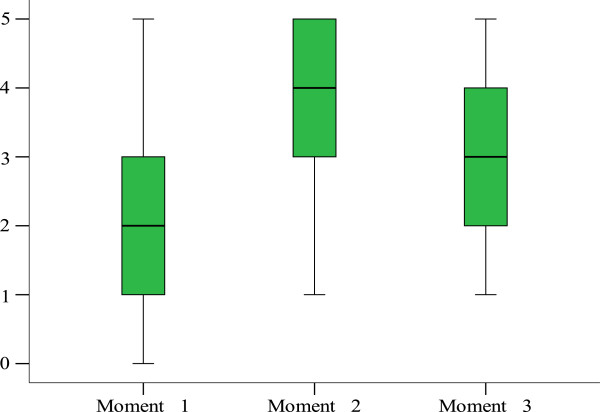
**Friedman’s variance results for diagnosis: median of successes variance over the three moments, Bolama Region, Guinea**-**Bissau, 2011.** Legend: Variations on the median of success in identification of the correct Diagnosis identification by CHWs, over the three moments of evaluation.

Friedman’s variance analysis showed an improvement of the median success rates from the first to the second moment of evaluation (*P* = 0.001, *n* = 22), though the differences registered from the second to the third moment were not statistically significant (*P* = 0.086, *n* = 22) (Figure 3). Cochrane’s Q test also showed statistically significant differences in several consultations, with multiple comparisons indicating a statistically significant improvement for moment 1 to moment 2 (*P* = 0.005, *n* = 22 for C1; *P* = 0.012, *n* = 22 for C3; and *P* = 0.001, *n* = 22 for C5). No statistical significant differences were found when comparing moments 2 and 3.

### Treatments

In the first moment the item Oral hydration with water was rarely prescribed (4%) - when recommended - but, after training this rate increased to 80%. Another finding is that the correct prescription *of* ORS hydration in the three moments was never above 60%, which means that, at best, 40% of the children were not prescribed the correct treatment when they presented with signs indicating its need. Regarding ‘no need for SRO hydration’ only 23% of the cases were correctly identified by the CHW in the first moment, which means that in 77% of cases the CHW prescribed a treatment that was not necessary. The correct decision rate improved after training (from 23% to 79%) though a decrease became patent 3 months after training (58%).

Friedman’s variance test showed significant differences for all items. Multiple comparisons reinforce the trends previously identified. The median number of correct identification for all items increased significantly from the first to the second moment for Hydration with water (*P* = 0.001, *n* = 22); SRO hydration (*P* <0.001, *n* = 22) and Send to hospital (*P* = 0.001, *n* = 22). Nevertheless, the median of correct identification of the item Send to hospital decreased significantly from the second to the third moment (*P* = 0.010, *n* = 22). The Cochran’s Q test results also showed significant statistical differences for all items. Multiple comparisons showed a statistically significant increase in the proportion of correctly identified items in the first transition (moment 1 to moment 2): Hydration with water (*P* = 0.028, *n* = 22); SRO hydration (*P* = 0.001, *n* = 22); and Send to hospital (*P* = 0.002, *n* = 22). When comparing moments 2 and 3, the proportion of correct item identification decreased significantly in two items: SRO hydration (*P* = 0.002, *n* = 22) and Send to hospital (*P* = 0.023, *n* = 22).

### Logistic regression model

Due to the small sample size (*N* = 22), results were limited to LR models based on a single explanatory variable (unadjusted results). Since there were only five female CHWs, the variable Sex was not considered in the model. Thus, the significance levels regarding the influence of the explanatory variables on the performance of the CHWs should be interpreted with care. However, some observed trends can be highlighted as they may reflect causes that influenced CHWs’ performance.

Younger CHWs tend to be more accurate in diarrhoeal diseases management (Table 1). A higher level of education seems to be related with better performance levels too (Tables 1 and 2). When comparing the number of training programmes attended and the CHW accuracy, no specific trend was found. However, *P* values found in one of the regressions suggest that CHWs with less training have a better performance. CHWs with less experience also seem to be more capable to better diagnose and treat diarrhoeal diseases. A higher level of theoretical knowledge obtained in the written test seems to have a positive influence upon the CHWs’ performance.

**Table 1:** **Results for the LR model applied for C1 considering the improvement in evacuations, Bolama Region, Guinea**-**Bissau, 2011**

**Variable**	**OR**	**95% CL**	***P***
Age	0.9	0.806 – 1.004	0.060
Level of education	0.678	1.021 – 2.756	0.041
Trainings (*n*)	0.875	0.668 – 1.146	0.332
Number of years as CHW	0.924	0.795 – 1.075	0.306
Theoretical knowledge before training	n/a	n/a	n/a
Theoretical knowledge after training	1.114	0.995 – 1.247	0.060

**Table 2:** **Results for the LR model applied for C5 considering the registered improvement in Diagnosis identification**, **Bolama Region**, **Guinea**-**Bissau**, **2011**

**Variable**	**OR**	**95% CL**	***P***
Age	0.909	0.814 – 1.015	0.090
Level of education	1.664	1.010 – 2.743	0.046
Trainings (*n*)	0.737	0.534 – 1.018	0.64
Number of years as CHW	0.898	0.770 – 1.047	0.171
Theoretical knowledge before training	1.061	0.985 – 1.143	0.120
Theoretical knowledge after training	1.053	0.989 – 1.121	0.108

## Discussion

The increase in theoretical knowledge after training indicates that CHWs were capable to understand the theoretical concepts presented to them. Similar results were found with CWHs in Senegal [12]. However, the research question in this study was to evaluate CHWs’ practice and assess how they understood the guidelines taught in the training courses as well. Standard procedures, flowcharts and guidelines can help to improve CHWs’ performance [24,25]. Nevertheless, the simple use of written guidelines is ineffective, as CHWs tend to stop using them over time [25,26]. Our results show that CHWs were able to understand theory and guidelines for medical practice within a limited period of time (the improvement registered from the first to the second moment), but those improvements vanished over time (3 months after).

CHWs improved significantly the correct identification of two identification-obligatory items from the first to the second moment, which proves that CHWs integrated the obligation of asking for Number of days with diarrhoea and Number of daily dejections transmitted in the training. However, focusing on obligatory items, as suggested during training, could have led to some negligence in recognizing subjective and harder to identify items. Competence-based training tend to improve a limited set of competencies [26], whereas evaluation methods tend to target the global learning experience. This can also justify why the identification rate for some items decreased from the first to the second moment.

Results obtained for Diagnosis show that training can help to improve CHWs’ competencies. The confusion between Severe dehydration and Acute dehydration registered before training was almost dissipated after training, which means that CHWs adhere to simple guidelines. These instruments seemed to be helpful to clarify doubts and help the CHWs choosing the correct diagnosis [25].

Both over-prescribing and under-prescribing were registered in Treatments. This can be related to two factors: under-prescription reflects the adaptation of CHWs to shortages in resources (medicines, for example) whereas over-prescription can be related to the existence of service fees that can lead to biased prescriptions intended to increase CHWs’ or health units’ revenues [25].

The small number of referrals to hospital can be explained by two reasons. First, the local presence of the referee doctor could make CHWs feel more confident about dubious cases, as they knew that the doctor could correct their errors later; second, in dubious cases, CHWs may have felt under pressure from families or communities to restrain from sending the patient to the hospital [27]. The cost-effectiveness of CHWs should be carefully considered because, on the one hand, their interventions can treat simple diseases at low cost in remote rural areas, therefore avoiding worsening clinical evolution and expensive evacuations and treatments but, on the other hand, bad prescriptions can lead to stock ruptures and catastrophic expenditures to families (when prescribing medicines or referrals with no indication). Through increased coverage and the provision of health care at low cost CHWs can contribute to improvements in health indicators [6,21]. However, supervision and support shall not remain underestimated, as a higher number of people become exposed to the risk of inadequate health care [26].

The results presented above point out that skills-based training loses its impact if regular supervision and support are not provided [24-27]. These follow-up activities are the more valuable when training is provided over short periods of time - because CHWs without formal education have greater difficulties in acquiring knowledge and skills [24].

As high levels of education seem to be correlated with better performances, the results support the idea that CHW programmes should include a minimum level of education as a selection criterion [6]. Literacy level also seems to be related to the capacity of retaining information, as CHW with higher levels of education tend to better retain knowledge, while those with less education lose skills over time [28]. Despite this criterion can be appellative to younger CHWs, they are not always accepted and respected by communities, which tend to prefer older people even if they are less educated [16].

CHWs who attend a lower number of trainings tend to be more effective - probably because training is provided by a variety of trainers, different contents, course organization and methodologies, which may create a sense of ambiguity. The changing on algorithms in follow-up trainings might also be responsible for confusing CHWs who attend multiple trainings [26,27].

The sample size constitutes one of the main limitations of the study. Statistical analysis was affected by the small level of participants, mainly when logistic regression models were performed. However, the trends observed are useful to understand some factors affecting CHWs’ performance. As the study only evaluated the CHWs of Bolama, the results cannot be extrapolated to the national reality of Guinea-Bissau. It should also be recognized that, by doing the study in a real environment, some skills taught in the training sessions were not evaluated for some of the CHWs. In addition, since CHWs were feeling observed, they might also have been more careful in their procedures [27].

## Conclusions

Training is crucial to health workers and has a particular importance for non-qualified workers, such as CHWs. The use of CHWs can be an alternative to a shortage (or mal-distribution) of qualified health professionals, but it is important to assess and highlight operational factors that can improve the performance of such programmes.

In this particular area, training, support and supervision play a fundamental role. The lack of continuous training, support and supervision might constitute a possible reason for the decrease in the performance of the CHWs over time. Continuous training is important since it helps refreshing past knowledge and practice, helping to identify and correct mistakes. Moreover, supervision and support help to ensure quality of care, as well as CHWs’ motivation.

National guidelines and training manuals could facilitate consistency in training information over time. At the time of this study (2011), guidelines for CHW training were being written, which can be a good start to improve the performance of these workers. Setting selection criteria can also be used as a strategy to improve CHWs’ performance. However, cultural questions should be addressed in order to avoid community rejection of younger and better educated CHWs.

Many countries adopted CHW programmes without monitoring and assessment of their impact, therefore disregarding the conditions for a good performance [26]. The implementation of these programmes should be always accompanied with assessments to identify the benefits and added value of interventions, as well as understanding the reasons for successes and failures [25]. Appropriate adaptations of programmes over time must also be considered and based on local level research and continuous learning [29]. As research in Guinea-Bissau is missing on this subject, the aim of this study was to evaluate the impact of training, and to suggest possible solutions to the problems identified. While a new programme is being prepared in Guinea-Bissau, we are pleased to see that selection criteria for CHWs were included, and a training manual is being prepared for distribution across all health regions. However, many questions about continuous training, supervision and support are still unanswered.

Further research should be conducted to explore other factors influencing CHWs’ performance, such as supervision, support, continuous training or even CHWs’ motivation and their integration within the health system. Clarification of roles and responsibilities, adequate incentives, systematic evaluation of CHW performance, joint supervisions and creation of effective linkages with formal health system are, among others, reported as activities that might contribute to this incorporation of CHW in the health system [29]. Research on these matters should also be focus of attention when implementing CHW programmes.

At a policy-making level, decision must balance existing resources and quality of care to be provided. Low income countries such as Guinea-Bissau investing in health programmes need to make a clear evaluation prior implementation on the best human resource strategy to be used. If CHW programmes need high investment on continuous training, supervision and support, training formal human resources may be initially expensive but more effective in the long term. A serious evaluation must be conducted to asses if countries shall rely on CHW or invest in qualified human resources for health groups.

Country and even local research must be conducted to drive decision-making on human resources for health. As several factors seem to affect CHWs performance, continuous learning and experimentation may help to find appropriate local adaptations to each programme [29]. Each country must look for the best solution for human resources chronicle shortages.

Quality of care and programmes’ effectiveness must be at the centre of decision-making rather than short-term economic considerations only. CHWs may be effective in providing some sort of health care if programmes are properly designed, implemented and based on existing evidence.

## Abbreviations

BHU: Basic health units; CHW: Community health workers; DTA: Diagnosis and treatment accuracy; LR: Logistic regression; HRB: Sanitary Region of Bolama; UNICEF: United Nations Children’s Fund; WHO: World Health Organization.

## Competing interests

The authors have no competing interests to declare.

## Authors’ contributions

SCL, AJC and BdeS drafted the manuscript. SCL and BdeS conducted the statistical analysis. All authors critically revised and approved the manuscript.

## Authors’ information

This article is a synopsis of the dissertation report of SCL, for the 3rd Master’s course in Health and Development, taught at Instituto de Higiene e Medicina Tropical – Universidade Nova de Lisboa. The study was also presented at the 13th World Congress of Public Health, held in Addis Ababa, Ethiopia, April 2012.
